# Fear of Falling, Anxiety, and Depressive Symptoms Among Older Adults in Bangladesh: Prevalence, Correlates, and Mediation Pathways

**DOI:** 10.1155/da/7793148

**Published:** 2026-07-20

**Authors:** Bijuriya Islam, Sagotom Subrota Barai, Most. Mrittika Khatun, Abir Hossain, Mortuja Mahamud Tohan, Satyajit Kundu, Tuhin Roy, Md. Ashfikur Rahman

**Affiliations:** ^1^ Development Studies, Social Science School, Khulna University, Khulna 9208, Bangladesh, ku.ac.bd; ^2^ Department of Public Administration, University of Dhaka, Dhaka 1000, Bangladesh, du.ac.bd; ^3^ School of General Education, BRAC University, Dhaka, Bangladesh, bracu.ac.bd; ^4^ Public Health, School of Medicine and Dentistry, Griffith University, Gold Coast, Queensland, 4222, Australia, griffith.edu.au; ^5^ Sociology Discipline, Social Science School, Khulna University, Khulna 9208, Bangladesh, ku.ac.bd; ^6^ Applied Social Sciences, Faculty of Health and Social Sciences, The Hong Kong Polytechnic University, HKSAR, Hong Kong, China, polyu.edu.hk

**Keywords:** anxiety, Bangladesh, depression, fear of falling, mental wellness, older adults

## Abstract

Fear of falling (FoF) is a common psychological burden among older adults, while FoF is closely linked to heightened anxiety and depressive symptoms, yet evidence from Bangladesh and South Asia remains inadequate. This study examined the mediated relationship between FoF, anxiety, and depressive symptoms among older adults in Bangladesh. A community‐based cross‐sectional survey was conducted with 251 participants using face‐to‐face structured interviews. Validated tools included the Falls Efficacy Scale–International (FES‐I‐16), Geriatric Depression Scale (GDS‐15), and Generalized Anxiety Disorder Scale (GAD‐7). Statistical analyses comprise descriptive statistics, analysis of variance (ANOVA) with effect size estimation, linear regression, and mediation analyses using bias‐corrected bootstrapping. The mean age of the older adults was 70.19 years, ranging from 60 to 85. A substantial proportion of participants (72.3%) reported having high concern about falling. Anxiety symptoms were more evenly distributed (33.9% minimal/mild, 26.3% moderate, and 39.8% high), while depressive symptoms were largely mild (57.8%), with fewer moderate (26.3%) and severe (15.9%) cases. Mediation analysis showed a significant total effect of FoF on depressive symptoms (*β* = 0.615, *p*  < 0.001). Anxiety symptoms partially mediated this relationship (indirect effect, *β* = 0.215, *p*  < 0.001), accounting for an about 35% of the total effect, while the direct effect remained significant (*β* = 0.400, *p*  < 0.001). FoF was directly associated with anxiety symptoms (*β* = 0.471, *p*  < 0.001), which in turn were positively associated with depressive symptoms (*β* = 0.457, *p*  < 0.001). Findings highlight FoF as a critical psychosocial factor associated with older mental health in Bangladesh. Integrating fall‐prevention strategies with psychosocial interventions may reduce anxiety and depressive symptoms, underscoring the need for context‐sensitive strategies in older populations.

## 1. Introduction

The global demographic shift toward older populations has heightened concern about the mental health and quality of life of older adults. Among mental health conditions, depressive symptoms and anxiety are one of the most common psychiatric disorders in later life, associated with increased morbidity, disability, and mortality [1]. In low‐ and middle‐income countries (LMICs) such as Bangladesh, the burden of older depressive symptoms is further compounded by limited healthcare resources, social isolation, and cultural practices that may exacerbate vulnerability [2]. Identifying modifiable psychosocial risk factors of these mental conditions is therefore critical for informing prevention and intervention strategies to improve mental health outcomes among the older population.

Falls and fear of falling (FoF) are closely related public health concerns among older populations. Globally, falls are recognized as the second leading cause of unintentional injury‐related mortality, accounting for approximately 684,000 deaths each year, most of which occur in LMICs [3]. Older adults aged 60 years and above experience the highest proportion of fatal fall‐related outcomes. In addition to mortality, falls impose a substantial morbidity burden, with an estimated 37.3 million fall‐related injuries requiring medical attention annually [3]. Beyond mortality, falls also impose a substantial morbidity burden, with an estimated 37.3 million cases each year requiring medical attention [3]. However, the consequences of falls extend beyond physical injury. FoF can restrict mobility, reduce confidence, and lead older adults to avoid daily activities. Such avoidance may contribute to physical deconditioning, functional decline, and an increased risk of future falls, creating a self‐reinforcing cycle of fear, avoidance, reduced activity, and further decline [4]. Falls and FoF are increasingly recognized in the existing literature not only as physical health concerns but also as problems with important psychosocial implications for older adults. Evidence shows that FoF is associated with reduced physical and mental functioning, activity restriction, increased fall risk, and poorer health‐related quality of life [5, 6]. FoF may reduce confidence in mobility, perceived independence, and participation in daily and social activities, which are central to psychological well‐being in later life [6, 7]. Fall‐related psychological concerns are also associated with anxiety among community‐dwelling older adults, while emerging evidence suggests that FoF may contribute to depressive symptoms directly or indirectly through reduced activity, social withdrawal, and lower quality of life [6–9]. Therefore, examining FoF alongside anxiety and depressive symptoms is important for identifying older adults who may be vulnerable to both physical limitations and psychological distress.

Anxiety and depressive symptoms are commonly reported mental health conditions among older adults worldwide, which represent a major public health concern among the older population. An estimated 14% of the population aged 70+ experiences a mental illness, which constitutes 6.8% of global disability [10]. Prevalence of depressive symptoms in older adults is around 35%, while that of anxiety disorder is 28%, with FoF being present among nearly half (50%) individuals [11]. A scoping review carried out with just South Asian countries has documented that in Bangladesh, depressive and anxiety disorders are especially common, affecting 55.5% and 55.7% of the elderly population, respectively; likewise, in Nepal, community‐dwelling older adults reported prevalence rates of 15.4% for depression, 18.1% for anxiety, 40.3% in India, and 11.4% in Sri Lanka [12]. FoF is also reported at a high level in Southeast Asia, varying between 21% and 88% [13]. Anxiety and depressive symptoms co‐occur frequently among older people; anxiety exacerbates depressive symptoms through excessive worry and rumination, while depressive symptoms augment anxiety through negative cognitive styles, resulting in a reduction of both mental health and quality of life.

This issue is particularly important for Bangladesh, where a rapid demographic transition is increasing the size and proportion of the older population. Older adults in Bangladesh often face poverty, chronic illness, limited access to older adults care, and inadequate social support, all of which may increase their vulnerability to both physical and mental health problems [14]. According to the Bangladesh Bureau of Statistics (BBS), the proportion of the population aged 60 years and above is projected to rise from approximately 8% at present to 22% by 2050; this corresponds to an increase from around 17 million older adults to nearly 45 million within the next three decades [15]. This demographic shift highlights the need for greater attention to aging‐related health challenges, including FoF, anxiety, and depressive symptoms among older adults in Bangladesh.

Despite the global recognition of FoF as a determinant of older mental health, Bangladesh‐specific evidence remains scarce. Cultural practices, limited older access, and high prevalence of comorbidities can further complicate the connection between FoF and depressive symptoms within this context as many older adults in Bangladesh live with multiple chronic health conditions that increase the vulnerability to physiological and psychological distress [16]. Moreover, anxiety may act as a mediating pathway, translating the fear into depressive symptoms. Understanding this mechanism is crucial for designing interventions that target both the psychological and behavioral dimensions of aging. With the Bangladesh‐specific cultural and social contexts in mind, these challenges will need to be tackled with a multidimensional dynamic approach considering the social situation, caregiving roles, and healthcare accessibility issues that the elderly population face [16]. This study, therefore, addresses a critical evidence gap by:

• Estimating the prevalence of FoF, anxiety, and depressive symptoms among older adults in Bangladesh.
• Examining the associated factors of anxiety and depressive symptoms with, after adjusting for other variables.
• Testing the mediation role of anxiety symptoms in the relationship between FoF and depressive symptoms.

By integrating prevalence estimates with mediation analysis, the study provides a nuanced understanding of older adult’s mental health in Bangladesh, offering insights that have policy implications. Findings will inform large‐scale screening, psychosocial interventions, and fall‐prevention programs, ultimately contributing to healthy aging trajectories in resource‐constrained settings. Although this study primarily focuses on the interrelationship among FoF, anxiety symptoms, and depressive symptoms, selected sociodemographic and health‐related characteristics were also considered because they may shape older adults’ vulnerability to physical limitations, psychological distress, and reduced quality of life [2, 17, 18].

## 2. Methods

### 2.1. Study Design, Setting, and Participants

This study employed a community‐based, cross‐sectional design to investigate the FoF, anxiety, and depressive symptoms among older adults in Bangladesh. The study was conducted in both urban and rural areas from Khulna Sadar upazila and different rural areas of Gopalganj in Bangladesh to capture diverse socioeconomic and cultural contexts in which older adults reside. The selection of Khulna Sadar upazila (urban) and rural areas of Gopalganj was intentional to ensure the representation of diverse socioeconomic and cultural contexts in which older adults live in Bangladesh. Khulna Sadar, being an urban setting, reflects greater access to health services, infrastructure, and social networks but also highlights challenges such as rapid urbanization, social isolation, and shifting family structures. In contrast, rural Gopalganj provides insights into communities where traditional family support systems remain stronger but where healthcare facilities, mental health resources, and awareness are often limited. By including both urban and rural sites, the study aimed to capture variations in lived experiences, health‐seeking behaviors, and the influence of cultural norms on mental health among older adults. This dual‐site approach strengthens the study’s ability to offer a more comprehensive understanding of the challenges faced by the elderly populations across Bangladesh. Participants were eligible for inclusion if they were aged 60 years old or above. This age cut‐off was selected because the study was conducted in Bangladesh, where individuals aged 60 years and above are recognized as older/senior citizens, even this threshold is also commonly used in national demographic reporting and aging‐related research in Bangladesh [17]. Eligibility criteria included permanent residence in the community for at least 1 year, ability to provide informed consent, and absence of severe cognitive impairment or acute medical illness that would preclude participation. Individuals with major psychiatric disorders such as bipolar disorders or dementia other than depressive symptoms or anxiety were excluded. Data was collected through structured, face‐to‐face interviews administered by authors.

### 2.2. Population, Sample Size, and Sampling Approach

The sample size was calculated using the single population proportion formula, based on prevalence of 50% for depressive symptoms (*p* = 0.5) among older adults in Bangladesh [2]. This conservative estimate was chosen to maximize statistical precision and ensure adequate power for subgroup and mediation analyses. Using a 95% confidence level (*z* = 1.96) and a margin of error of 5% (*d* = 0.05), the minimum required sample size was computed as follows: 

n=z2×p1−pd2=1.962×0.5×10.5−0.052=384.16384.≈

After adding a 10% allowance for potential non‐response or incomplete data, the target sample size was approximately 422 participants. However, due to field‐level constraints, eligibility criteria, and the availability of older adults during data collection, 255 eligible participants were approached. Of these, 251 completed the survey and had complete data for analysis, yielding a response rate of 98.4% (251/255 × 100), which is considered statistically enough for community‐based studies. Although the final analytical sample was smaller than the initially estimated target, the sample was adequate for the planned descriptive, bivariate, and regression‐based analyses. Participants were recruited through a multistage cluster sampling approach to ensure representativeness across regions. A multistage cluster sampling strategy was employed to ensure representativeness while maintaining feasibility in data collection. In the first stage, two districts of Bangladesh were purposively selected to capture diverse socioeconomic and cultural contexts: Khulna Sadar upazila (urban) and rural areas of Gopalganj. Within each district, clusters were defined at the community level (wards in urban areas and villages in rural areas). In the second stage, households with older adults (aged 60 years and above) were identified within each cluster using household listings and local records. Finally, in the third stage, one eligible older adult per household was randomly selected for an interview. This multistage design allowed for systematic coverage of both urban and rural populations, reduced logistical challenges, and minimized sampling bias. By clustering households and then randomly selecting participants within clusters, the approach balanced efficiency with representativeness, ensuring that the sample reflected variations in the socioeconomic status, healthcare access, and cultural practices across the study areas.

### 2.3. Measurement Instruments and Variables

Depressive symptoms were measured using the 15‐item Geriatric Depression Scale (GDS‐15), a widely validated screening tool specifically designed for older adults, with binary (yes/no) responses that capture affective and cognitive aspects of depressive symptoms; higher scores reflect greater depressive symptoms [19]. Of the 15 items on the Geriatric Depression Scale (GDS‐15), 10 are scored positively to indicate the presence of depressive symptoms, while the remaining five items (numbers 1, 5, 7, 11, and 13) are reverse‐scored, with negative responses reflecting depressive symptoms. The total score ranged from 0 to 15, with higher scores denoting greater symptom severity. A score of 0–4 is generally considered within the normal range (depending on age, education, and clinical complaints), 5–8 suggests mild, 9–11 indicates moderate, and 12–15 reflects severe depressive symptoms [20].

Anxiety was assessed using the Generalized Anxiety Disorder Scale (GAD‐7), a brief, 7‐item self‐report questionnaire that evaluates the frequency of anxiety symptoms over the past 2 weeks on a four‐point Likert scale; it has been validated in both primary care and general populations, showing excellent reliability and construct [21]. Each item was scored from 0 to 3 based on symptom frequency over the past 2 weeks, giving a total score range of 0–21. Cut‐off points classify severity: 0–4 (minimal), 5–9 (mild), 10–14 (moderate), ≥15 (severe) [22].

FoF was assessed using the Falls Efficacy Scale–International (FES‐I, 16 items), which measures the level of concern about falling during a range of physical and social activities; higher scores indicate greater FoF, and the instrument has demonstrated strong cross‐cultural validity in older adults [23]. The FES‐I (16) is a brief and easily administered instrument designed to assess the degree of concern about falling across 16 everyday social and physical activities, both within and outside the home, regardless of whether the individual actually performs the activity. Responses are rated on a four‐point Likert scale ranging from 1 = not at all concerned to 4 = very concerned, with higher scores indicating greater FoF [23]. The scores were grouped as follows: low concern [16–19], moderate concern [20–27], and high concern (≥28), with higher scores reflecting a greater FoF.

Other variables were selected based on prior empirical evidence and contextual considerations specific to older populations in Bangladesh and similar settings [2, 16, 18, 24]. Sociodemographic characteristics included age, sex, marital status, education level, religion, personal income, living condition, employment status, area of residence, and economic dependency. Lifestyle variables encompassed religious activities, physical exercise, and smoking. Psychosocial factors such as social support, living arrangements, and community participation were included. The primary psychosocial construct of interest, FoF (FES‐I, 16), was treated as the main exposure variable, while anxiety symptoms (GAD‐7) were considered as a potential mediator in the association between FoF and depressive symptoms (GDS‐15). Variables were selected to ensure comprehensive coverage of the demographic, behavioral, and psychosocial domains.

### 2.4. Statistical Analyses and Model Diagnostics

Descriptive statistics were first computed to summarize the sociodemographic, behavioral, and psychosocial characteristics of the study participants. Continuous variables were reported as means with standard deviations (SDs), while categorical variables were expressed as frequencies and percentages. Group differences were examined using analysis of variance (ANOVA) and independent sample *t*‐tests for continuous outcomes and chi‐square tests for categorical variables. In addition to statistical significance, effect sizes (*η* ^2^ and partial *η* ^2^) were calculated for ANOVA models to quantify the magnitude of group differences, with thresholds interpreted as small (*η* ^2^ ≈ 0.01), medium (*η* ^2^ ≈ 0.06), and large (*η* ^2^ ≥ 0.14) [25].

To identify factors associated with anxiety and depressive symptoms, multivariable linear regression models were fitted, adjusting for sociodemographic, FoF, and other lifestyle covariates. Standardized beta coefficients (*β*), 95% confidence intervals (CI), and *p*‐values were reported. In addition to the main regression results, supplementary tables are provided to document the diagnostic and supporting analyses that ensure the robustness of our findings. While (Table S1) reports collinearity statistics (VIF and tolerance) to confirm that multicollinearity was not a concern among predictors. Table S2 presents overall model fit indices (*R* ^2^, adjusted *R* ^2^, AIC, BIC, RMSE, and *F*‐test) to demonstrate the adequacy of the regression model. Finally, (Table S3) provides the full set of regression coefficients, including categorical contrasts, before collapsing into the simplified presentation of the regression results. Together, these supporting information enhance transparency, reproducibility, and confidence in the validity of the reported associations. Furthermore, the *Q*–*Q* plot (Figure S1) conducting such analysis allowed us to verify that the regression model’s residuals did not deviate substantially from normality. This strengthens confidence in the validity of the regression estimates reported in the main results, since violations of normality could bias standard errors, CIs, and hypothesis tests. Thus, the *Q*–*Q* plot provides visual evidence that the assumptions of linear regression were appropriately met, supporting the conclusion that our model represents a good fit for the data and its trustworthiness.

The hypothesized mediation pathway was tested using Jamovi 2.6.22, with bias‐corrected bootstrap resampling (5000 iterations), while other analyses were executed using IBM SPSS.25 and STATA.17. Anxiety (GAD‐7) was specified as the mediator in the relationship between the FoF (FES‐I, 16) and depressive symptoms (GDS‐15). Indirect, direct, and total effects were estimated, with mediation considered significant if the 95% CI excluded zero. In addition, exploratory moderation analyses were performed to assess whether sociodemographic variables (e.g., sex and marital status) modified the strength of associations. The internal consistency of all psychometric scales was evaluated using Cronbach’s alpha coefficients, with values ≥0.70 considered acceptable. Reliability analyses confirmed satisfactory internal consistency for the FES‐I,16, GAD‐7, and GDS‐15 in this sample. All statistical tests were two‐tailed, and a *p*‐value  < 0.05 was considered statistically significant. Sociodemographic and health‐related variables were included as contextual covariates to describe the study population and to account for potential background differences in FoF, anxiety symptoms, and depressive symptoms.

## 3. Results

### 3.1. General Characteristics of the Participants

Table 1 summarizes the general characteristics of the 251 study participants. Females constituted 52.6% of the sample, while 47.4% were males. A higher proportion of respondents resided in urban areas (59.4%) compared with those in rural areas (40.6%).

**Table 1:** General characteristics of the study participants.

Variables	Frequency (*N*)	Percent (%)
Sex of the respondents		
Female	132	52.6
Male	119	47.4
Age of the respondents		
Mean age (70.19); range (60–85)	—	—
Early seniors (60–67 years)	88	35.1
Mid seniors (68–75 years)	112	44.6
Late seniors (≥76 years)	51	20.3
Area of residence		
Rural	102	40.6
Urban	149	59.4
Religion		
Hindu	30	12.0
Islam	221	88.0
Education level		
No education	54	21.5
Primary or secondary	125	49.8
Higher and above	72	28.7
Marital status		
Married	158	62.9
Widowed	93	37.1
Economic dependency		
Dependent on others	133	53.0
Self‐dependent	118	47.0
Personal income		
No	146	58.2
Yes	105	41.8
Employment status		
Employed full time	21	8.4
Never employed	89	35.5
Retired	141	56.2
Living condition		
Alone	48	19.1
With family	203	80.9
Religious activities		
Never	23	9.2
Often	46	18.3
Regular	127	50.6
Sometimes	55	21.9
Physical exercise		
Never	52	20.7
Sometimes	78	31.1
Often	50	19.9
Regular	71	28.3
Smoking		
No	204	81.3
Yes	47	18.7
Fear of falling (FoF)		
Low concern	21	8.4
Medium concern	48	19.3
High concern	182	72.3
Anxiety symptoms		
Minimal/mild anxiety	85	33.9
Moderate anxiety	66	26.3
High anxiety	100	39.8
Depressive symptoms		
No/mild	145	57.8
Moderate	66	26.3
Severe	40	15.9

**Figure 1:**
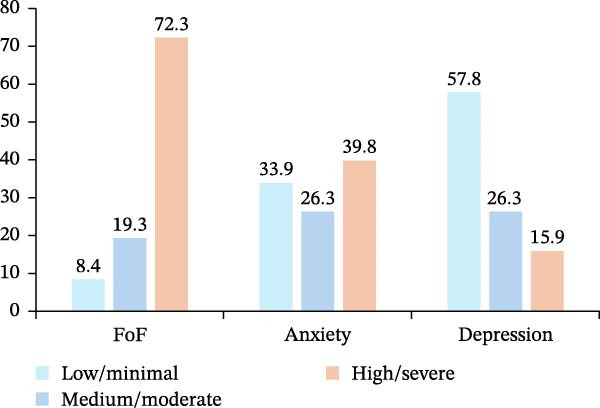
Prevalence of psychological disorders.

Figure 1 illustrates the severity distribution of FoF, anxiety, and depressive symptoms among older adults in Bangladesh. FoF shows a striking concentration in the high/severe category (72.3%), indicating that most participants experience substantial concern about falling during daily activities. Anxiety levels are more evenly distributed, with 33.9% reporting minimal/mild symptoms, 26.3% moderate, and 39.8% high anxiety. In contrast, depressive symptoms are predominantly mild, with 57.8% of participants in the no/mild category and fewer reporting moderate (26.3%) or severe (15.9%) depressive symptoms.

**Figure 2:**
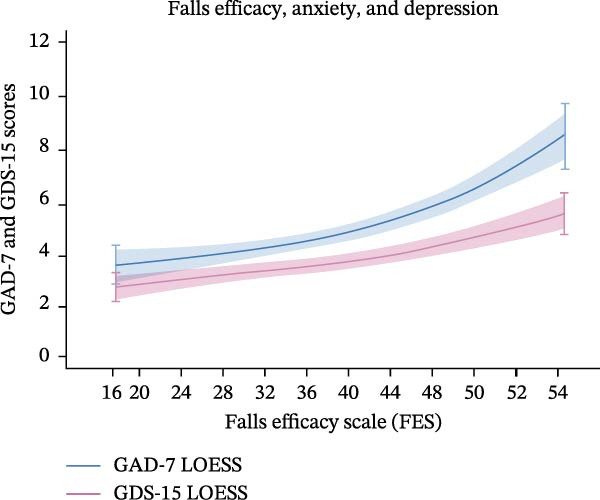
Falls efficacy scale scores and their psychological correlations.

Figure 2 shows the relationship between the FoF and psychological distress. LOESS‐smoothed curves show a positive association between Falls Efficacy Scale (FES) scores and both GAD‐7 and GDS‐15 scores, indicating that greater FoF (higher FES scores, reflecting lower confidence in avoiding falls) was correlated with increased symptoms of anxiety and depression. This upward trend persisted across the full FES range (16–54), with overlapping CIs supporting the robustness of the association.

As shown in Table 2, the FES‐I (16) had a mean score of 46.02 (SD = 11.17; 95% CI: 44.63–47.41), indicating a high level of concern about falling among participants. The scale demonstrated good internal consistency (Cronbach’s *α* = 0.845). The Geriatric Depression Scale (GDS‐15) showed a mean score of 7.26 (SD = 4.01; 95% CI: 6.76–7.76), suggesting mild to moderate depressive symptoms with acceptable reliability (*α* = 0.749). The Generalized Anxiety Disorder scale (GAD‐7) yielded a mean score of 12.22 (SD = 5.53; 95% CI: 11.53–12.91), corresponding to moderate anxiety levels and demonstrating good internal consistency (*α* = 0.821). Overall, all three instruments showed adequate to good reliability, supporting their use in assessing fall‐related concerns, depressive symptoms, and anxiety in the study population.

**Table 2:** Scale scores and internal consistency estimates.

Scales name	Mean (std. deviation)	95% CI	Cronbach’s alpha
Falls efficacy scale (FES‐I, 16)	46.02 (11.17)	44.63–47.41	0.845
Geriatric depressive scale (GDS‐15)	7.26 (4.01)	6.76–7.76	0.749
Generalized anxiety scale (GAD‐7)	12.22 (5.53)	11.53–12.91	0.821

The distribution of GAD‐7 and GDS‐15 scores across all variables is shown in Table 3. Participants with no formal education reported higher anxiety and depressive symptoms scores than those with primary or secondary education (GAD‐7: *p* = 0.04; GDS‐15: *p* = 0.03), with small to moderate effect sizes (*η* ^2^ = 0.06–0.07). Respondents who were economically dependent had significantly higher scores on both scales compared with independent participants (GAD‐7: *p* = 0.02, *d* = 0.25; GDS‐15: *p* = 0.01, *d* = 0.28). Similarly, participants without personal income showed higher scores on both scales than those with income (GAD‐7: *p* = 0.03, *d* = 0.24; GDS‐15: *p* = 0.02, *d* = 0.26). Individuals living alone reported higher GAD‐7 and GDS‐15 scores than those living with family (*p* = 0.04 and *p* = 0.03, respectively). In addition, respondents who engaged with religious activities and physical exercise also showed higher anxiety and depressive symptom scores compared to participants who never engaged in these activities (*η* ^2^ ≈ 0.05–0.06). No statistically significant differences in anxiety or depressive symptoms scores were found by sex, area of residence, religion, marital status, employment status, or smoking (all *p*  > 0.05).

**Table 3:** Subgroup analysis of GAD‐7 and GDS‐15 scores.

Variable category	Mean GAD‐7 (SD)	*p*‐Value	Effect size	Mean GDS‐15 (SD)	*p*‐Value	Effect size
Sex of the respondents						
Female	12.8 (5.4)	0.11	*d* = 0.23	7.5 (4.0)	0.17	*d* = 0.20
Male	11.6 (5.7)			7.0 (4.1)		
Age of the respondents						
Early seniors (60–67 years)	11.91 (5.45)	0.309	*η* ^2^ = 0.010	7.63 (4.00)	0.273	*η* ^2^ = 0.009
Mid seniors (68–75 years)	11.96 (5.79)			6.83 (4.17)		
Late seniors (≥76 years)	13.33 (5.01)			7.59 (3.62)		
Area of the respondents						
Rural	12.5 (5.6)	0.32	*d* = 0.15	7.4 (4.2)	0.28	*d* = 0.14
Urban	11.9 (5.5)			7.1 (3.9)		
Religion						
Hindu/others	12.1 (5.4)	0.67	*d* = 0.05	7.2 (4.0)	0.71	*d* = 0.04
Muslim	12.3 (5.7)			7.3 (4.1)		
Level of education (ANOVA)						
No education	13.0 (5.8)	0.004	*η* ^2^ = 0.06	8.0 (4.3)	0.003	*η* ^2^ = 0.07
Primary	12.0 (5.3)			7.2 (4.0)		
Secondary	11.5 (5.2)			6.8 (3.8)		
Marital status						
Married	12.0 (5.5)	0.21	*d* = 0.12	7.1 (4.0)	0.19	*d* = 0.13
Widowed/other	12.7 (5.6)			7.6 (4.2)		
Economic dependency						
Dependent	12.8 (5.7)	0.002	*d* = 0.25	7.8 (4.2)	0.001	*d* = 0.28
Independent	11.4 (5.1)			6.7 (3.9)		
Personal income						
No	12.7 (5.6)	0.003	*d* = 0.24	7.7 (4.1)	0.002	*d* = 0.26
Yes	11.2 (5.2)			6.6 (3.8)		
Employment status						
Employed FT	11.0 (5.0)	0.09	*d* = 0.21	6.8 (3.9)	0.12	*d* = 0.19
Other	12.4 (5.6)			7.4 (4.1)		
Living status						
Alone	13.5 (5.9)	0.004	*d* = 0.27	8.1 (4.4)	0.003	*d* = 0.29
With family	11.9 (5.4)			7.1 (4.0)		
Religious activities (ANOVA)						
Never	13.2 (5.8)	0.005	*η* ^2^ = 0.05	8.1 (4.4)	0.004	*η* ^2^ = 0.06
Regular	11.8 (5.3)			7.0 (3.9)		
Sometimes/often	12.3 (5.5)			7.3 (4.0)		
Exercise (ANOVA)						
Never	13.4 (5.9)	0.05	*η* ^2^ = 0.05	8.1 (4.4)	0.04	*η* ^2^ = 0.06
Regular	11.7 (5.2)			7.0 (3.9)		
Sometimes/often	12.2 (5.5)			7.3 (4.0)		
Smoking						
No	12.1 (5.4)	0.41	*d* = 0.09	7.2 (4.0)	0.38	*d* = 0.10
Yes	12.9 (5.8)			7.6 (4.2)		
Fear of falling (FoF)						
Low concern	2.1 (1.4)	0.001	*η* ^2^ = 0.28	1.8 (1.2)	0.001	*η* ^2^ = 0.24
Medium concern	4.3 (2.0)			3.2 (1.8)		
High concern	6.8 (2.5)			5.1 (2.3)		

After adjustment for all variables, FoF emerged as a significant determinant of both depressive and anxiety symptoms (Table 4). Compared with participants reporting low concern, those with high concern about falling had positive association with depressive (*β* = 4.83, 95% CI: 0.73 to 8.94,*p* = 0.021), and anxiety symptoms (*β* = 9.47, 95% CI: 2.46 to 16.49, *p* = 0.008). Living status was strongly associated with mental health outcomes. Participants living alone reported significantly higher levels of both depressive (*β* = 2.71, 95% CI: 1.73 to 3.69, *p* < 0.001) and anxiety symptoms (*β* = 3.68, 95% CI: 2.01to 5.34, *p* < 0.001). Older adults who engaged in physical exercise reported significantly lower depressive and anxiety symptoms compared to those who did not. Analyses showed that depressive (*β* = −1.86, SE = 0.38, 95% CI = −1.11 to −2.60, *p*  < 0.001) and anxiety (*β* = −2.08, SE = 0.65, 95% CI = −1.80 to −3.35, *p* = 0.001) were less among those who perform any kind of physical activity. Being economically dependent was significantly associated with higher depressive symptoms (*β* = 1.74, 95% CI: 1.00 to 2.47, *p* < 0.001). Widowed or other marital status was also associated with increased depressive symptoms (*β* = 1.22, 95% CI: 0.43 to 2.01, *p* = 0.003). In addition, smoking use was significantly associated with higher depressive symptoms (*β* = 1.40, 95% CI: 0.45 to 2.36, *p* = 0.004).

**Table 4:** Multivariable regression analysis of depressive and anxiety symptoms.

Variable	Depressive symptoms (GDS‐15)					Anxiety symptoms (GAD‐7)				
	Coefficient (*β*)	Std. error	*t*	95% CI	*p*‐Value	Coefficient (*β*)	Std. error	*t*	95% CI	*p*‐Value
Concerned related to falls										
Low concern (RC)	—	—	—	—	—	—	—	—	—	—
Medium concern	0.55	2.15	0.26	−3.69,4.79	0.797	7.21	3.67	1.96	−0.04, 14.46	0.051
High concern	4.83	2.08	2.32	0.73, 8.94	0.021	9.47	3.56	2.66	2.46, 16.49	0.008
Sex of the respondents										
Male (RC)	—	—	—	—	—	—	—	—	—	—
Female	0.03	0.37	0.07	−0.70, 0.75	0.946	−0.48	0.63	−0.76	−1.72, 0.76	0.448
Area of residence										
Rural (RC)	—	—	—	—	—	—	—	—	—	—
Urban	−0.03	0.38	−0.07	−0.77, 0.71	0.946	0.09	0.64	0.15	−1.17, 1.34	0.884
Level of education										
No formal education (RC)	—	—	—	—	—	—	—	—	—	—
Primary or secondary	0.10	0.47	0.21	−0.83, 1.03	0.832	0.88	0.80	1.09	−0.70, 2.46	0.275
Higher education	0.43	0.53	0.82	−0.60, 1.47	0.412	1.14	0.90	1.27	−0.63, 2.91	0.207
Marital status										
Married (RC)	—	—	—	—	—	—	—	—	—	—
Widowed and others	1.22	0.40	3.03	0.43, 2.01	0.003	1.05	0.69	1.52	−0.31, 2.40	0.129
Economic dependency										
Independent (RC)	—	—	—	—	—	—	—	—	—	—
Dependent on others	1.74	0.37	4.63	1.00, 2.47	<0.001	1.15	0.64	1.79	−0.12, 2.41	0.075
Personal income										
No (RC)	—	—	—	—	—	—	—	—	—	—
Yes	0.05	0.37	0.14	−0.68, 0.78	0.891	−0.30	0.63	−0.47	−1.55, 0.94	0.639
Employment status										
Currently employed (RC)	—	—	—	—	—	—	—	—	—	—
Never employed	−0.90	0.70	−1.28	−2.29, 0.49	0.202	1.38	1.20	1.14	−0.99, 3.75	0.254
Retired	−1.15	0.68	−1.68	−2.49, 0.20	0.095	0.54	1.17	0.46	−1.76, 2.83	0.646
Living status										
With family/others (RC)	—	—	—	—	—	—	—	—	—	—
Alone	2.71	0.50	5.46	1.73, 3.69	<0.001	3.68	0.84	4.33	2.01, 5.34	0.001
Perform physical exercise										
No (RC)	—	—	—	—	—	—	—	—	—	—
Yes	−1.86	0.38	−4.92	−1.11, −2.60	<0.001	−2.08	0.65	3.22	−1.80, −3.35	0.001
Smoking										
No (RC)	—	—	—	—	—	—	—	—	—	—
Yes	1.40	0.48	2.90	0.45, 2.36	0.004	1.27	0.83	1.53	−0.36, 2.90	0.127

As shown in Table 5, it presents the mediation analysis examining the indirect and total effects of fall‐related concern (FES‐I) on depressive symptoms (GDS‐15) through anxiety symptoms (GAD‐7). All CIs were estimated using bias‐corrected bootstrap methods. A significant indirect effect of fall‐related concern on depressive symptoms via anxiety was observed (estimate = 0.077, SE = 0.012, 95% CI: 0.056–0.104, *β* = 0.215, *z* = 6.30, *p* < 0.001), indicating that anxiety symptoms partially mediated the relationship between FoF and depressive symptoms. Both component paths were statistically significant: higher fall‐related concern was associated with increased anxiety (FES ⇒ GAD‐7: *β* = 0.471, *p* < 0.001), and higher anxiety was associated with increased depressive symptoms (GAD‐7 ⇒ GDS‐15: *β* = 0.457, *p* < 0.001). The direct effect of fall‐related concern on depressive symptoms remained significant after accounting for anxiety (estimate = 0.144, 95% CI: 0.107–0.182, *β* = 0.400, *p* < 0.001), indicating partial mediation. The total effect of fall‐related concern on depressive symptoms was also significant (estimate = 0.221, 95% CI: 0.186–0.254, *β* = 0.615, *p* < 0.001), demonstrating a substantial overall association. Overall, these findings indicate that the FoF influences depressive symptoms both directly and indirectly through increased anxiety, with anxiety accounting for a meaningful proportion of the total effect.

**Table 5:** Mediation analysis of the association between fall‐related concern, anxiety, and depressive symptoms.

Indirect and total effects								
Type	Effect	Estimate	SE	95% CI^a^		*β*	*z*	*p*
				Lower	Upper			
Indirect	FES ⇒ GAD ⇒ GDS	0.0772	0.0123	0.0555	0.104	0.215	6.30	<0.001
Component	FES ⇒ GAD	0.2332	0.0275	0.1777	0.283	0.471	8.47	<0.001
	GAD ⇒ GDS	0.3312	0.0352	0.2630	0.394	0.457	9.42	<0.001
Direct	FES ⇒ GDS	0.1435	0.0174	0.1073	0.182	0.400	8.25	<0.001
Total	FES ⇒ GDS	0.2207	0.0179	0.1863	0.254	0.615	12.34	<0.001

*Note:* Betas are completely standardized effect sizes.

^a^Confidence intervals computed with method: bias corrected bootstrap.

**Figure 3:**
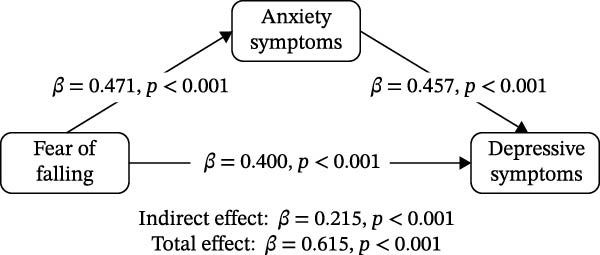
Structural relationships among falls efficacy, anxiety, and depressive symptoms.

The path diagram in Figure 3 depicts the directional associations among FoF, anxiety, and depressive symptoms using standardized regression coefficients. FoF demonstrated a direct positive association with anxiety symptoms (*β* = 0.23), indicating that higher self‐reported FoF was linked to elevated anxiety symptoms. Anxiety, in turn, showed a significant direct effect on depressive symptoms (*β* = 0.33). Additionally, falls efficacy exhibited a modest direct association with depressive symptoms (*β* = 0.14). These findings suggest a partial mediation pathway, whereby the influence of FoF on depressive symptoms is both direct and indirectly mediated through anxiety symptoms. The positive direction of all paths challenges conventional expectations and may reflect complex psychosocial dynamics or measurement artifacts in self‐perceived physical competence among individuals experiencing psychological distress.

## 4. Discussion

The global prevalence of FoF was found to be higher in developing countries at (53.40%), where developed ones remained 46.7% [13], while the parallel trend has been reported in some Asian countries, specifically Malaysia, Singapore, Thailand, and Vietnam, where the prevalence was found to be 21.6%–88.2% [26]. One cross‐sectional study was carried in Bangladesh where the prevalence was reported 67.5% [27] our findings are consistent with patterns of FoF. The extent of depressive symptoms and anxiety is also consistent with other Bangladeshi studies [2, 16, 18].

Our findings suggest that anxiety and depressive symptoms and FoF among older adults are intricately interconnected rather than distinct categories, which is aligned with the global literature [28]. Consistent with our findings, prior studies from Europe, North America, China, and some south Asian countries show that FoF leads to depressive symptoms that started with anxiety, intensifying psychological disorders [29, 30]. The observed association among FoF, anxiety symptoms, and depressive symptoms may be explained through a fear‐avoidance and psychosocial restriction pathway. It is because FoF is not only a consequence of previous falls but also an independent psychological concern that may reduce confidence, restrict mobility, and limit engagement in routine and outdoor activities. Systematic evidence suggests that FoF is associated with activity restriction, poorer physical and mental functioning, increased risk of future falls, and reduced health‐related quality of life among older adults [5, 6, 31]. When older adults avoid walking, household tasks, or community participation because of the FoF, this may initially appear protective; however, prolonged avoidance can contribute to physical deconditioning, reduced autonomy, and further loss of confidence. FoF is also closely related to anxiety, as persistent worry about falling, injury, or loss of independence may intensify psychological distress [8, 29]. At the same time, reduced mobility and avoidance of social activities may decrease social interaction and increase isolation or loneliness, which can further contribute to depressive symptoms [7]. Recent evidence also indicates that FoF and falls are associated with depressive symptoms among community‐dwelling older adults, while depression may mediate the relationship between FoF and poorer quality of life [24, 32]. Therefore, FoF, anxiety, and depressive symptoms should not be viewed as separate conditions but as interconnected experiences that may reinforce one another in later life.

Older adults in Bangladesh experience anxiety and psychological difficulties due to insufficient public facilities incorporating uneven pavements and poor residential conditions, which are intricately associated with FoF [33]. Studies conducted in the USA and China showed that anxiety is a primary mediator, aligning with the outcomes drawn from the USA and research from China [34, 35], the present study demonstrates anxiety as a central mediator, which revealed anxiety‐related distress among aging individuals dealing with physical constraints encompassing those in Bangladesh. Despite consistency, our study found a more pronounced effect of the mediating factor than that in Western nations. Mental health literacy and aging intervention remain insufficient in Bangladesh, resulting in long‐term emotional distress, which intrinsically remains unaddressed and later transforms into depressive symptoms overtime [36, 37]. Financial reliance further exacerbates the emotional distress of the older individuals; our findings revealed that in accordance with studies elsewhere [38, 39]. Conversely, Bangladeshi older individuals feel insecure due to the financial strain that spans over generations, further triggering their depressive symptoms, whereas European states provide pension security, which lessens poor mental health problems [40, 41].

Beyond the primary pathway linking FoF, anxiety, and depressive symptoms, selected contextual factors also showed associations with psychological distress and are briefly discussed. Living alone, lack of regular physical exercise, and smoking were also associated with heightening anxiety and/or depressive symptoms in this study, consistent with previous evidence elsewhere [42–44]. Living alone may increase psychological vulnerability through social isolation, reduced emotional support, loneliness, and concerns about illness, falls, and unmet care needs [42, 43, 45]. However, co‐residence is not always protective, as family conflict, financial stress, unemployment, divorce, or caregiving strain may limit emotional support [46, 47]. Regular physical exercise may reduce psychological distress by improving mood, physical functioning, self‐efficacy, and social engagement [48, 49]. Smoking may contribute to depressive symptoms through nicotine dependence, withdrawal‐related mood disturbance, and its bidirectional relationship with poor mental health [44, 50]. Evidence pointed out that smoking cessation improves depression and anxiety further supports integrating tobacco cessation into older‐adult mental health care [51, 52].

This study has several notable strengths. It employed validated psychometric instruments (FES‐I, 16, GAD‐7, and GDS‐15) with demonstrated reliability in older populations, and all scales were culturally adapted into Bangla to enhance contextual relevance. The inclusion of diverse sociodemographic, behavioral, and health‐related variables allowed for a comprehensive examination of the interplay between FoF, anxiety, and depressive symptoms, while the use of advanced statistical techniques (ANOVA, regression, and mediation analyses with effect size estimation) strengthened the robustness of findings, and it could be a strong baseline study for further comprehensive investigation. Nevertheless, certain limitations should be acknowledged. The achieved sample size (*n* = 251), though adequate for medium‐effect detection, was lower than the initially calculated target, potentially reducing statistical power for subgroup analyses. The cross‐sectional design precludes causal inference, and reliance on self‐reported measures may introduce a recall or social desirability bias. Furthermore, the study was conducted in a single regional context, which may limit generalizability to other settings in Bangladesh or similar contexts. Despite these constraints, the findings provide valuable insights into psychosocial determinants of older mental health and highlight pathways for future longitudinal and intervention‐based research.

### 4.1. Potential Future Implications

The findings suggest that policy responses for older adults in Bangladesh should move beyond a narrow focus on physical fall prevention and adopt an integrated approach that addresses both fall risk and mental health. Because FoF is common in later life and is closely linked with anxiety‐related psychological concerns, routine elderly care should include brief screening for FoF, anxiety, and depressive symptoms in primary care and community settings. Early identification would allow timely referral and support before the psychological distress becomes more severe [8, 13, 53].

At the service‐delivery level, we strongly believe that Bangladesh would benefit from community‐based, multifactorial fall‐prevention programmes that combine balance and gait training, home‐safety assessment and modification, medication review, caregiver education, and psychosocial support. This is consistent with current international guidance, which recommends opportunistic case‐finding, comprehensive fall‐risk assessment, and tailored multidomain interventions for older adults, including in lower‐resource settings [54, 55]. A coordinated policy framework across the health, housing, and social support sectors is also needed. Improving housing safety, neighborhood accessibility, and community support for older adults may reduce both physical vulnerability and the persistent insecurity that sustains the FoF. In this sense, interventions should be embedded within broader healthy‐aging strategies that emphasize person‐centered, community‐based, and financially accessible care for older people [3].

Furthermore, Bangladesh should come forward to integrating fall‐prevention strategies with psychosocial screening and anxiety management within older care services, which could be crucial for reducing depressive symptoms and improving overall mental well‐being among older adults in low‐resource settings. Strengthening community‐based support systems and improving housing safety can reduce vulnerability among older adults. Training healthcare providers in older mental health and expanding social protection schemes are critical to address financial dependency and limited social support. Finally, a coordinated policy framework across health, housing, and social welfare sectors is essential to ensure sustainable interventions.

## 5. Conclusion

This study provides empirical evidence from Bangladesh that FoF is a significant psychosocial stressor among older adults and is strongly associated with depressive symptoms. The findings demonstrate that anxiety plays a partial mediating role in this relationship, indicating that FoF contributes to depressive symptoms both directly and indirectly through heightened anxiety symptoms. These results highlight the interconnected nature of physical vulnerability and psychological distress in later life. Addressing FoF without considering its anxiety‐related pathways may, therefore, limit the effectiveness of interventions.

## Author Contributions

Md. Ashfikur Rahman and Mortuja Mahamud Tohan did the formal analysis. Data collection and extraction were carried out by Bijuriya Islam and Sagotom Subrota Barai. The design and conceptualization were carried out by Bijuriya Islam and Md. Ashfikur Rahman. Original manuscript was drafted by Bijuriya Islam, Sagotom Subrota Barai, Most. Mrittika Khatun, Abir Hossain, and Md. Ashfikur Rahman. Satyajit Kundu, Tuhin Roy, Mortuja Mahamud Tohan, and Md. Ashfikur Rahman reviewed extensively. Md. Ashfikur Rahman supervised and validated the study.

## Funding

No funding was received for this study.

## Disclosure

Md. Ashfikur Rahman and Mortuja Mahamud Tohan take all responsibility and liability for the analysis. All authors have read and approved the final version of the manuscript for submission.

## Ethics Statement

This research was approved by the Ethical Clearance Committee of Khulna University Research and Innovation Centre (RIC); the number of ethical approvals is KUEC: 2025‐11‐156. Verbal consent from all participants was received before the interview.

## Conflicts of Interest

The authors declare no conflicts of interest.

## Supporting Information

Additional supporting information can be found online in the Supporting Information section.

## Supporting information


**Supporting Information** The supporting analyses supported the robustness and validity of the regression findings. Collinearity diagnostics indicated no evidence of multicollinearity among the independent variables, with all variance inflation factor (VIF) values ranging from 1.03 to 1.13 and tolerance values exceeding 0.88. The overall regression model demonstrated a good fit to the data (*R* = 0.780, *R* ^2^ = 0.608, adjusted *R* ^2^ = 0.576; *F* = 18.9, *p* < 0.001), explaining approximately 61% of the variance in depressive symptoms. In the adjusted model, fear of falling remained a strong positive predictor of depressive symptoms, while marital status, economic dependency, living status, physical inactivity, and smoking/betel leaf use were also significantly associated with depression. Diagnostic plots further showed that model residuals were approximately normally distributed and randomly dispersed, indicating that the assumptions of linear regression were adequately met and supporting the reliability of the reported estimates.

## Data Availability

The data can be made available upon request.
