# Supplementary material for: Fear of Falling, Anxiety, and Depressive Symptoms Among Older Adults in Bangladesh: Prevalence, Correlates, and Mediation Pathways
Source: Depress Anxiety. 2026 Jul 20;2026:7793148. doi: 10.1155/da/7793148 (PMC13383248; doi:10.1155/da/7793148)
Supplement: Supplementary file 1 — Supporting Information The supporting analyses supported the robustness and validity of the regression findings. Collinearity diagnostics indicated no evidence of multicollinearity among the independent variables, with all variance inflation factor (VIF) values ranging from 1.03 to 1.13 and tolerance values exceeding 0.88. The overall regression model demonstrated a good fit to the data (R = 0.780, R 2 = 0.608, adjusted R 2 = 0.576; F = 18.9, p < 0.001), explaining approximately 61% of the variance in depressive symptoms. In the adjusted model, fear of falling remained a strong positive predictor of depressive symptoms, while marital status, economic dependency, living status, physical inactivity, and smoking/betel leaf use were also significantly associated with depression. Diagnostic plots further showed that model residuals were approximately normally distributed and randomly dispersed, indicating that the assumptions of linear regression were adequately met and supporting the reliability of the reported estimates. [file DA-2026-7793148-s001.docx]

| **Supplementary Table S1.** Collinearity Statistics. Variance inflation factors (VIF) and tolerance values for all predictors included in the regression analysis. | | |
| --- | --- | --- |
|  | **VIF** | **Tolerance** |
| FES_Total | 1.13 | 0.882 |
| Sex of the Respondents | 1.04 | 0.960 |
| Area of the Respondents | 1.05 | 0.956 |
| Religion | 1.03 | 0.969 |
| Education Level | 1.04 | 0.965 |
| Marital Status | 1.09 | 0.914 |
| Economic Dependency | 1.07 | 0.938 |
| Personal Income | 1.03 | 0.971 |
| Employment Status | 1.05 | 0.952 |
| Living Status | 1.09 | 0.917 |
| Religious Activities | 1.04 | 0.957 |
| Exercise | 1.05 | 0.957 |
| Smoking Beat Leaf | 1.08 | 0.924 |

| **Supplementary Table S2.** Model Fit Measures. Summary of overall model fit indices (R, R², Adjusted R², AIC, BIC, RMSE) and F‑test results for the regression model estimated with N = 251 | | | | | | | | | | |
| --- | --- | --- | --- | --- | --- | --- | --- | --- | --- | --- |
|  | | | | | | | **Overall Model Test** | | | |
| **Model** | **R** | **R²** | **Adjusted R²** | **AIC** | **BIC** | **RMSE** | **F** | **df1** | **df2** | **p** |
| 1 | 0.780 | 0.608 | 0.576 | 1215 | 1289 | 2.50 | 18.9 | 19 | 231 | <.001 |
| Note. Models estimated using sample size of N=251 | | | | | | | | | | |

| **Supplementary Table S3.** Model Coefficients for GDS15_Total. Regression coefficients, standard errors, t‑values, p‑values, standardized estimates, and 95% confidence intervals for predictors of GDS15_Total scores. | | | | | | | |
| --- | --- | --- | --- | --- | --- | --- | --- |
|  | | | | | | **95% Confidence Interval** | |
| **Predictor** | **Estimate** | **SE** | **t** | **p** | **Stand. Estimate** | **Lower** | **Upper** |
| Interceptᵃ | 2.7538 | 1.3672 | 2.014 | 0.045 |  |  |  |
| FES_Total | 0.1533 | 0.0167 | 9.154 | <.001 | 0.4272 | 0.33524 | 0.5192 |
| Sex of the Respondents |  |  |  |  |  |  |  |
| 2 – 1 | 0.2884 | 0.3436 | 0.839 | 0.402 | 0.0720 | -0.09698 | 0.2409 |
| Areas of Living |  |  |  |  |  |  |  |
| 2 – 1 | -0.2786 | 0.3508 | -0.794 | 0.428 | -0.0695 | -0.24199 | 0.1029 |
| Religion |  |  |  |  |  |  |  |
| 2 – 1 | 0.0530 | 0.5240 | 0.101 | 0.919 | 0.0132 | -0.24438 | 0.2708 |
| Education Level |  |  |  |  |  |  |  |
| 2 – 1 | 0.1723 | 0.4316 | 0.399 | 0.690 | 0.0430 | -0.16921 | 0.2552 |
| 3 – 1 | 0.2082 | 0.4803 | 0.433 | 0.665 | 0.0520 | -0.18419 | 0.2881 |
| Marital Status |  |  |  |  |  |  |  |
| 2 – 1 | 0.9538 | 0.3730 | 2.557 | 0.011 | 0.2380 | 0.05461 | 0.4214 |
| Economic dependency: |  |  |  |  |  |  |  |
| 2 – 1 | -1.3601 | 0.3518 | -3.866 | <.001 | -0.3394 | -0.51237 | -0.1664 |
| Personal income |  |  |  |  |  |  |  |
| 2 – 1 | 0.4103 | 0.3437 | 1.194 | 0.234 | 0.1024 | -0.06662 | 0.2714 |
| Employment status |  |  |  |  |  |  |  |
| 2 – 1 | -0.7622 | 0.6495 | -1.173 | 0.242 | -0.1902 | -0.50954 | 0.1291 |
| 3 – 1 | -0.9703 | 0.6357 | -1.526 | 0.128 | -0.2421 | -0.55468 | 0.0704 |
| Living status |  |  |  |  |  |  |  |
| 2 – 1 | -2.7609 | 0.4564 | -6.050 | <.001 | -0.6890 | -0.91334 | -0.4646 |
| Religious activities |  |  |  |  |  |  |  |
| 2 – 1 | -0.5643 | 0.7003 | -0.806 | 0.421 | -0.1408 | -0.48515 | 0.2035 |
| 3 – 1 | -0.1654 | 0.6008 | -0.275 | 0.783 | -0.0413 | -0.33664 | 0.2541 |
| 4 – 1 | 0.3520 | 0.6651 | 0.529 | 0.597 | 0.0878 | -0.23920 | 0.4149 |
| Exercise |  |  |  |  |  |  |  |
| 2 – 1 | -0.5703 | 0.4830 | -1.181 | 0.239 | -0.1423 | -0.37978 | 0.0951 |
| 3 – 1 | 0.4439 | 0.5306 | 0.837 | 0.404 | 0.1108 | -0.15008 | 0.3716 |
| 4 – 1 | 1.7104 | 0.5022 | 3.406 | <.001 | 0.4268 | 0.17989 | 0.6737 |
| Smoking beat leaf |  |  |  |  |  |  |  |
| 2 – 1 | 0.9089 | 0.4569 | 1.989 | 0.048 | 0.2268 | 0.00218 | 0.4514 |
| ᵃ Represents reference level | | | | | | | |


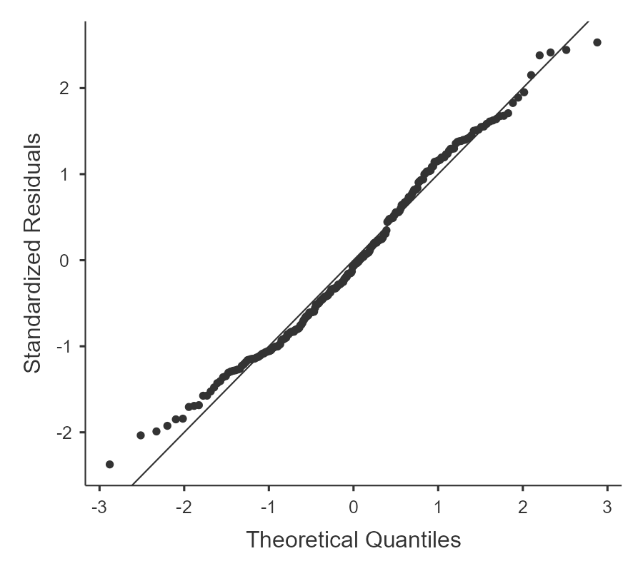

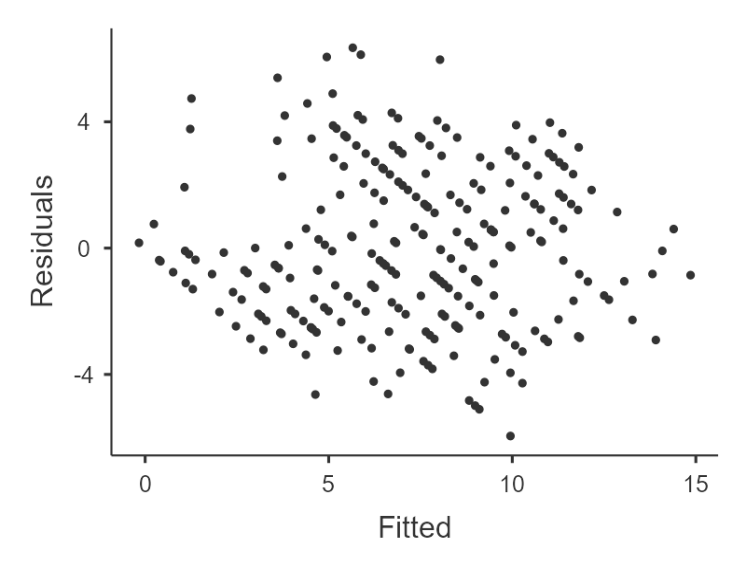


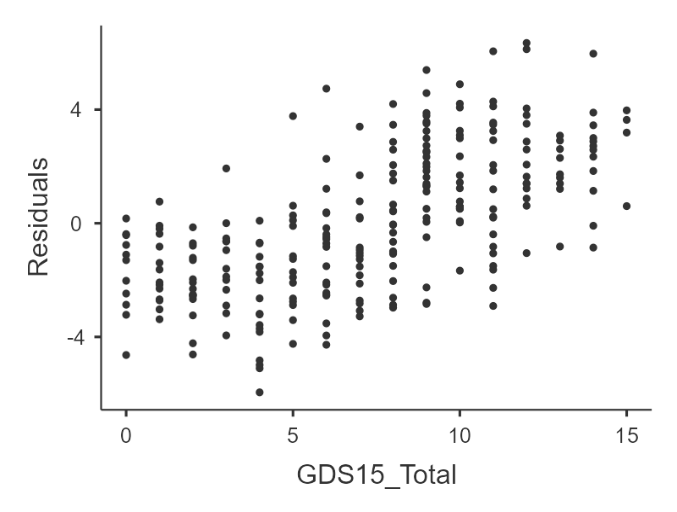

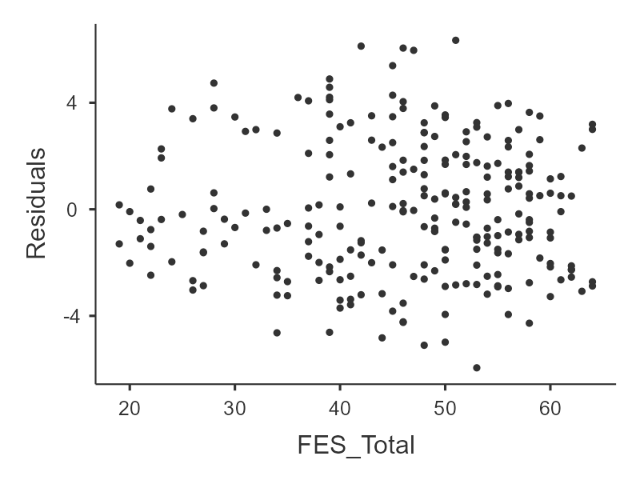


**Supplementary Figure S1.** Q–Q Plot normality assessment of residuals from the regression model using a quantile–quantile plot.

The Q–Q plot indicates that residuals approximate normality, supporting the validity of regression assumptions and the robustness of the results presented in Table 4.
